# Supplementary material for: Mirabegron displays anticancer effects by globally browning adipose tissues
Source: Nat Commun. 2023 Nov 22;14:7610. doi: 10.1038/s41467-023-43350-8 (PMC10665320; doi:10.1038/s41467-023-43350-8)
Supplement: Supplementary file 1 — Supplementary Infomation [file 41467_2023_43350_MOESM1_ESM.pdf]

Supplementary Information

Mirabegron displays anticancer effects by globally browning adipose tissues

Xiaoting Sun[1,2#], Wenhai Sui[3#], Zepeng Mu[4], Sisi Xie[5], Jinxiu Deng[5], Sen Li[5], Takahiro Seki[2], Jieyu Wu[2], Xu Jing[2,6], Xingkang He[7], Yangang Wang[4], Xiaokun Li[1], Yunlong Yang[5*], Ping Huang[8*], Minghua Ge[6*], and Yihai Cao[2*]

[1]Oujiang Laboratory (Zhejiang Lab for Regenerative Medicine, Vison and Brain Health), School of Pharmaceutical Science, Wenzhou Medical University, Wenzhou, China

[2]Department of Microbiology, Tumor and Cell Biology, Karolinska Institutet, 171 65 Solna, Sweden

[3]The Key Laboratory of Cardiovascular Remodeling and Function Research, Chinese Ministry of Education, Chinese National Health Commission and Chinese Academy of Medical Sciences, The State and Shandong Province Joint Key Laboratory of Translational Cardiovascular Medicine, Department of Cardiology, Qilu Hospital of Shandong University, 250012 Jinan, China

[4]Department of Endocrinology, Affiliated Hospital of Medical College Qingdao University, Qingdao, China

[5]Department of Cellular and Genetic Medicine, School of Basic Medical Sciences, Fudan University, 200032 Shanghai, China

[6]Department of Head and Neck Surgery, Center of Otolaryngology-Head and Neck Surgery, Zhejiang Provincial People's Hospital, People's Hospital of Hangzhou Medical College, Hangzhou, China

[7]Department of Gastroenterology, Sir Run Run Shaw Hospital, Zhejiang University Medical School, Hangzhou 310016, China

[8]Department of Pharmacy, Zhejiang Provincial People's Hospital, People's Hospital of Hangzhou Medical College, Hangzhou, China; Key Laboratory of Endocrine Gland Diseases of Zhejiang Province, China

Key words: Cancer, metabolism, brown adipose tissue, mirabegron, glycolysis

Running title: Mirabegron inhibits tumor growth by metabolic reprogramming

[#]These authors contributed equally

*Correspondence, galley proofs and reprint requests should be primarily addressed to:
Yunlong Yang, Ph.D., Department of Cellular and Genetic Medicine, School of Basic Medical Sciences, Fudan University, 200032 Shanghai, China. E-mail: yunlongyang@fudan.edu.cn; Ping Huang, M.D., Department of Pharmacy, Zhejiang Provincial People's Hospital, People's

Hospital of Hangzhou Medical College, Hangzhou, China; Key Laboratory of Endocrine Gland Diseases of Zhejiang Province, China. E-mail: huangping@hmc.edu.cn; Minghua Ge, M.D., Department of Head and Neck Surgery, Center of Otolaryngology-Head and Neck Surgery, Zhejiang Provincial People's Hospital, People's Hospital of Hangzhou Medical College, Hangzhou, China. Tel: (+86)-571-85239988, E-mail: gemingh@163.com; Yihai Cao, M.D., Ph.D., Department of Microbiology, Tumor and Cell Biology, Karolinska Institutet, 171 77 Stockholm, Sweden. Tel: (+46)-8-5248 7596, Fax: (+46)-8-33 13 99, E-mail: yihai.cao@ki.se

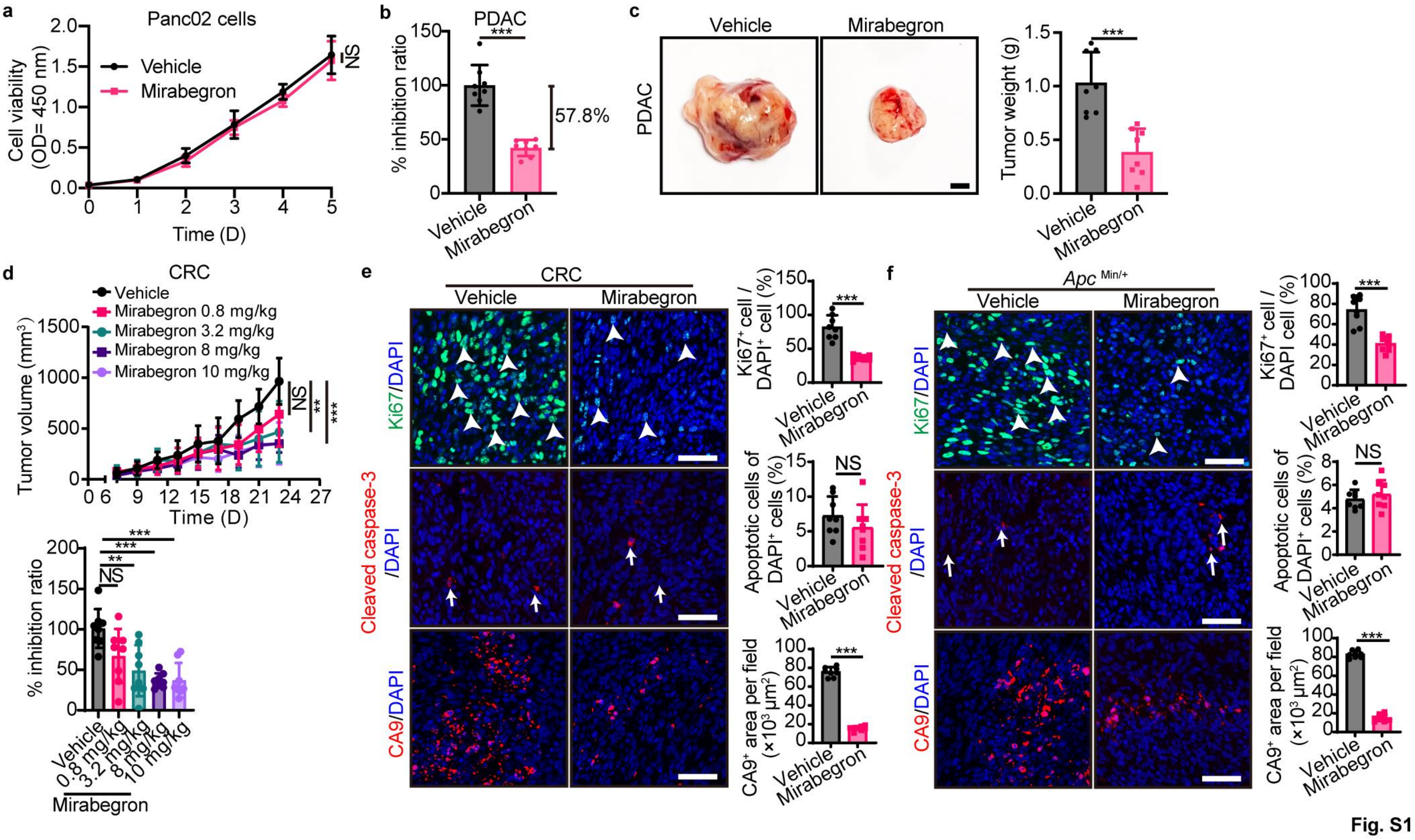

Fig. S1 Mirabegron inhibits tumor growth in vivo but not in vitro.

a. Tumor cell proliferation of vehicle- and mirabegron-treated Panc02 cells (n = 5 samples per group). **b.** Tumor inhibition ratios of vehicle- and mirabegron-treated PDAC tumor-bearing mice (n = 8 mice per group). **c.** Representative photographs and weights of tumor in vehicle- and mirabegron-treated PDAC tumor-bearing mice (n = 8 mice per group). Scale bar, 5 mm. **d.** Tumor growth and tumor inhibition ratios of vehicle- and 0.8, 3.2, 8, 10 mg/kg mirabegron-treated CRC tumor-bearing mice (n = 8 mice per group). **e.** Immunofluorescence staining of $Ki67^+$ proliferating cells (green), cleaved-caspase 3^+ apoptotic cells (red), and $CA9^+$ hypoxic area (red) of CRC tumors. Tissues were counterstained with DAPI (blue). Arrows and arrowheads point to their respective positive signals. Scale bar, 50 μm. Quantifications of $Ki67^+$ signal, cleaved-caspase 3^+ signal, and $CA9^+$ signal (n = 8 random fields per group). **f.**

Immunofluorescence staining of $Ki67^+$ proliferating cells (green), cleaved-caspase 3^+ apoptotic cells (red), and $CA9^+$ hypoxic area (red) of polyps in vehicle- and mirabegron-treated *Apc*$^{Min/+}$ mice. Tissues were counterstained with DAPI (blue). Arrows and arrowheads point to their respective positive signals. Scale bar, 50 µm. Quantifications of $Ki67^+$ signal, cleaved-caspase 3^+ signal, and $CA9^+$ signal (n = 8 random fields per group). Statistical analysis was performed using two-sided unpaired t-test (a-c, e-f) and one-way ANOVA test (d). NS, not significant; $*P < 0.05$; $**P < 0.01$; $***P < 0.001$. Data presented as mean ± SD. Each experiment was repeated at least three times and the representative experiment was shown (a-f). Source data are provided as a Source Data file.

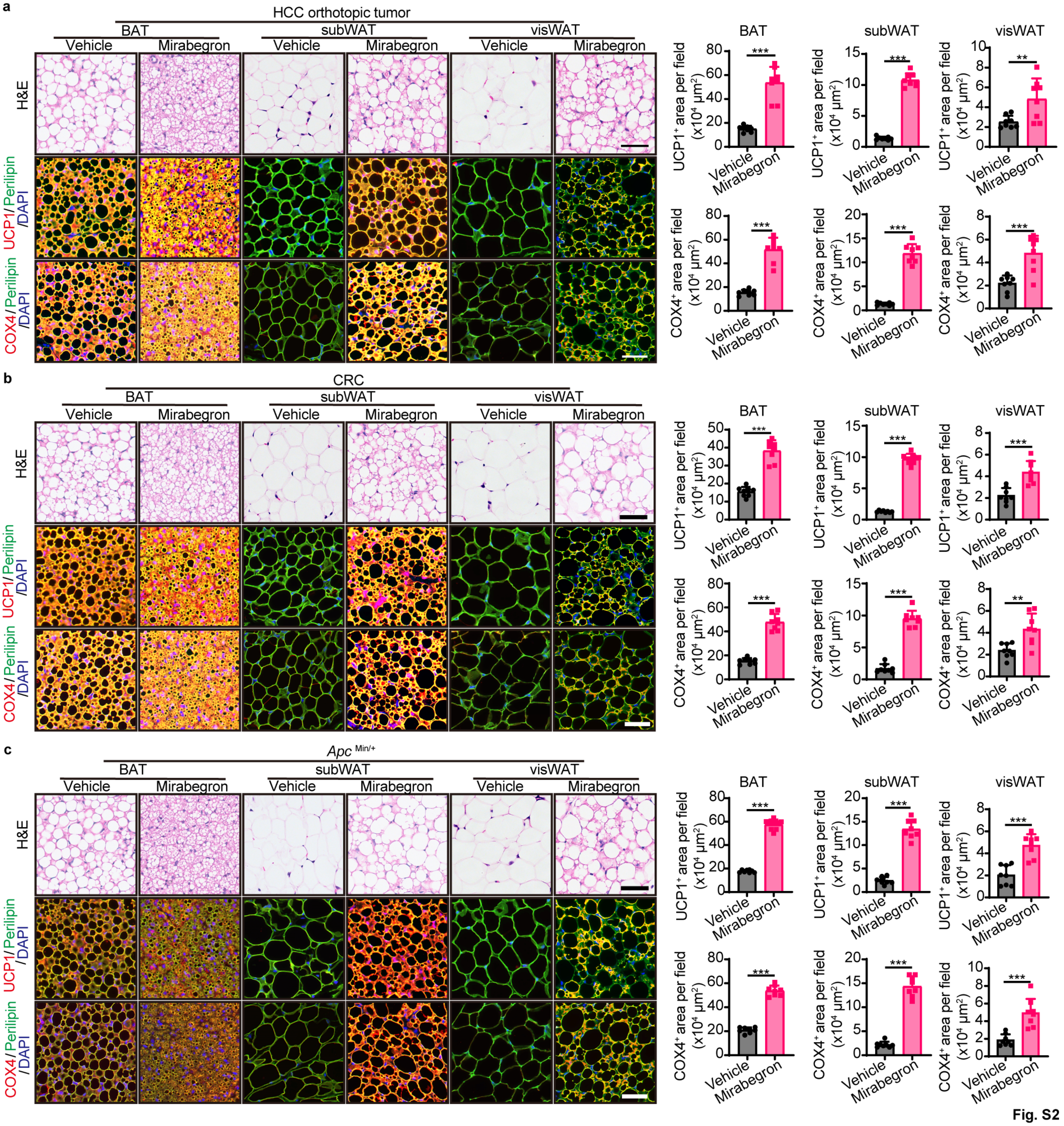

Fig. S2 Mirabegron-induced adipose tissue browning in tumor-bearing mice.

a-c. H&E histological staining and immunofluorescence staining of UCP1 (red), COX4 (red), and Perilipin (green) of adipose depots in vehicle- and mirabegron-treated orthotopic HCC tumor-bearing mice, CRC tumor-bearing mice, and *Apc*$^{Min/+}$ mice. Tissues were counterstained with DAPI (blue). Scale bar, 50 μm. Quantifications of UCP1^{+} signal and COX4^{+} signal (n = 8 random fields per group). Statistical analysis was performed using two-sided unpaired t-test (a-c). NS, not significant; **P* < 0.05; ***P* < 0.01; ****P* < 0.001. Data presented as mean ± SD. Each experiment was repeated at least three times and the representative experiment was shown (a-c). Source data are provided as a Source Data file.

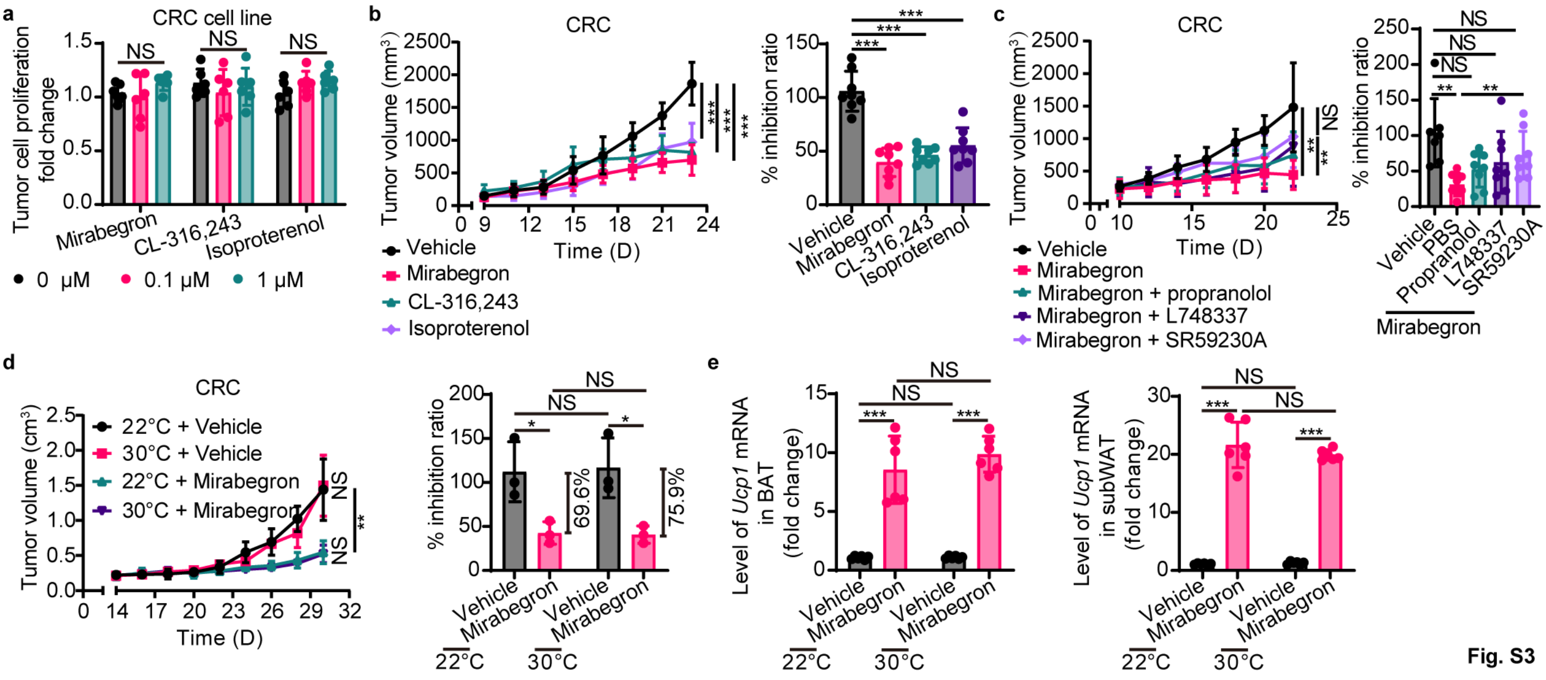

Fig. S3

Fig. S3 β3-AR agonist-triggered tumor suppression.

a. Tumor cell proliferation of vehicle-, mirabegron-, CL-316, 243-treated MC-38 cells (n = 6 samples per group). **b.** Tumor growth and inhibition ratios of vehicle-, mirabegron-, CL-316, 243-, or isoproterenol-treated CRC tumor-bearing mice (n = 8 mice per group). **c.** Tumor growth and inhibition ratios of mirabegron-treated CRC tumor-bearing mice receiving propranolol, L-748,337, or SR59230A (n = 7-8 mice per group). **d.** Tumor growth and tumor inhibition ratios of vehicle- and mirabegron-treated PDAC under 30 °C and 22 °C (n = 3 mice per group). **e.** *Ucp1* mRNA level of BAT and subWAT in vehicle- and mirabegron-treated tumor-bearing mice under 30 °C and 22 °C (n = 6 samples per group). Statistical analysis was performed using two-sided unpaired t-test (e) and one-way ANOVA test (a-d). NS, not significant; **P < 0.01; ***P < 0.001. Data presented as mean ± SD. Each experiment was repeated at least three times and the representative experiment was shown (a-e). Source data are provided as a Source Data file.

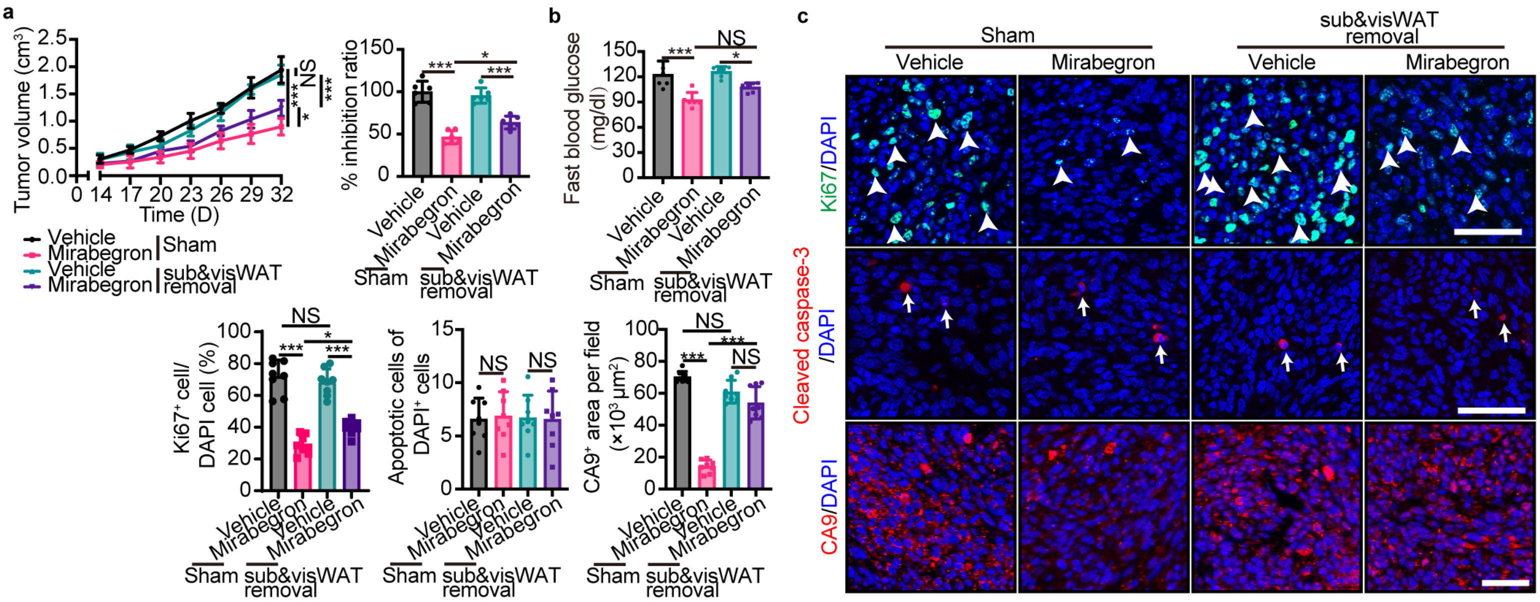

Fig. S4

Fig. S4 Removal of WATs partially ablates mirabegron-triggered tumor suppression.

a. PDAC tumor growth and inhibition ratios of sham and WATs (subWAT and visWAT) removal tumor-bearing mice with or without mirabegron treatment (n = 8 mice per group). **b.** Fast blood glucose levels of sham and WATs removal tumor-bearing mice with or without mirabegron treatment (n = 8 mice per group). **c.** Immunofluorescence staining of Ki67$^+$ proliferating cells (green), cleaved-caspase 3$^+$ apoptotic cells (red), and CA9$^+$ hypoxic area (red) of PDAC tumors. Tissues were counterstained with DAPI (blue). Arrows and arrowheads point to their respective positive signals. Scale bar, 50 μm. Quantifications of Ki67$^+$ signal, cleaved-caspase 3$^+$ signal, and CA9$^+$ signal (n = 8 random fields per group). Statistical analysis was performed using one-way ANOVA test (a-c). NS, not significant; **P* < 0.05; ***P* < 0.01; ****P* < 0.001. Data presented as mean ± SD. Each experiment was repeated at least three times and the representative experiment was shown (a-c). Source data are provided as a Source Data file.

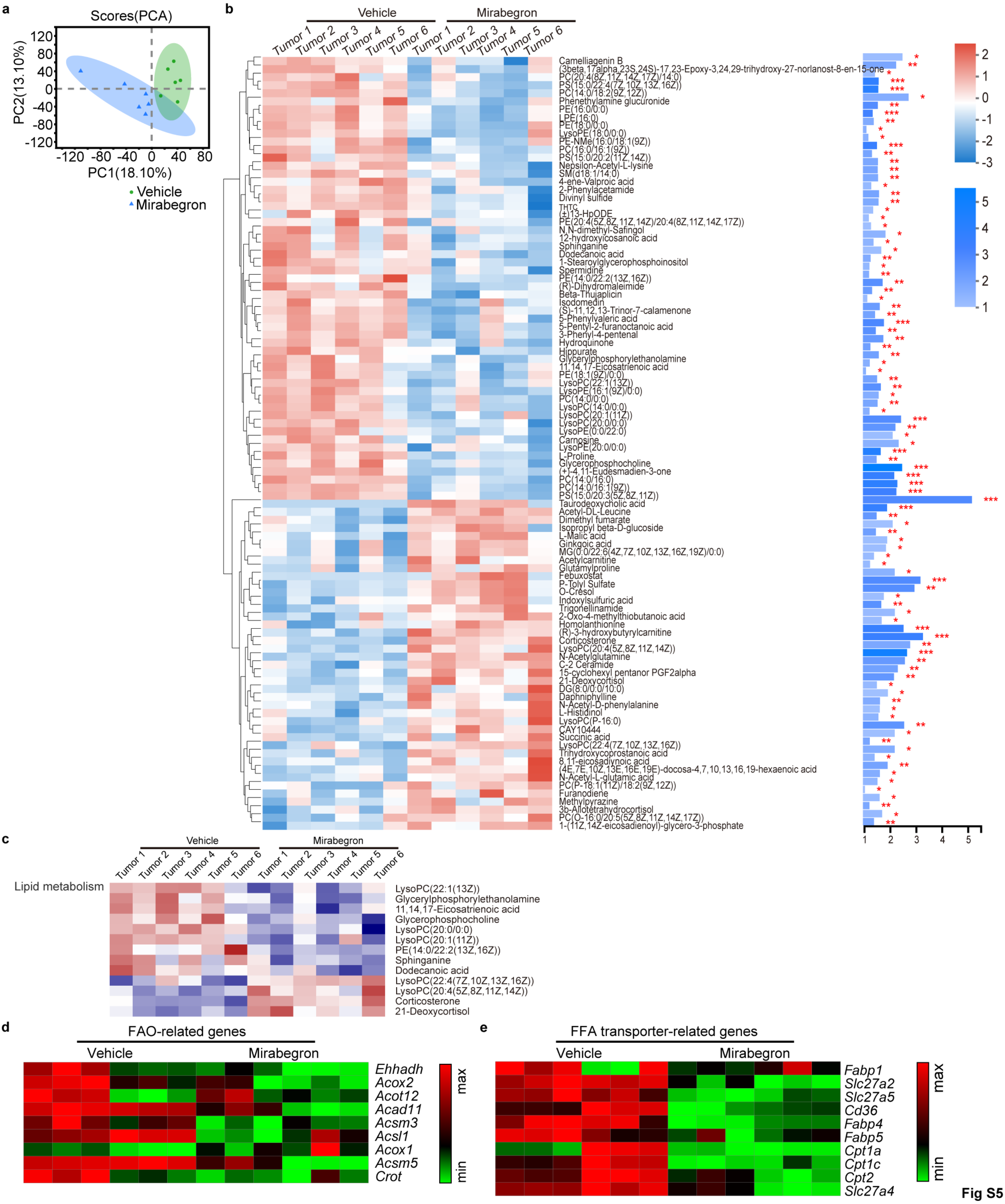
a
Scores(PCA)
PC2(13.10%)
PC1(18.10%)
Vehicle
Mirabegron
b
Vehicle
Mirabegron
Tumor 1
Tumor 2
Tumor 3
Tumor 4
Tumor 5
Tumor 6
Camelliagenin B
PS(15:0/22:4(7Z,10Z,13Z,16Z))
PC(14:0/18:2(9Z,12Z))
Phenethylamine glucuronide
PE(16:0/0:0)
LPE(16:0)
PE(18:0/0:0)
LysoPE(18:0/0:0)
PE-NMe(16:0/18:1(9Z))
PC(16:0/16:1(9Z))
PS(15:0/20:2(11Z,14Z))
Nepsilon-Acetyl-L-lysine
SM(d18:1/14:0)
4-ene-Valproic acid
2-Phenylacetamide
Divinyl sulfide
THTC
(±)13-HpODE
N,N-dimethyl-Safingol
12-hydroxyicosanoic acid
Sphinganine
Dodecanoic acid
1-Stearoylglycerophosphoinositol
Spermidine
PE(14:0/22:2(13Z,16Z))
(R)-Dihydromaleimide
Beta-Thujaplicin
Isodomedin
(S)-11,12,13-Trinor-7-calamenone
5-Phenylvaleric acid
5-Pentyl-2-furanoctanoic acid
3-Phenyl-4-pentenal
Hydroquinone
Hippurate
Glycerylphosphorylethanolamine
11,14,17-Eicosatrienoic acid
PE(18:1(9Z)/0:0)
LysoPC(22:1(13Z))
LysoPE(16:1(9Z)/0:0)
PC(14:0/0:0)
LysoPC(14:0/0:0)
LysoPC(20:1(11Z))
LysoPC(20:0/0:0)
LysoPE(0:0/22:0)
Carnosine
LysoPE(20:0/0:0)
L-Proline
Glycerophosphocholine
(+)-4,11-Eudesmadien-3-one
PC(14:0/16:0)
PC(14:0/16:1(9Z))
PS(15:0/20:3(5Z,8Z,11Z))
Taurodeoxycholic acid
Acetyl-DL-Leucine
Dimethyl fumarate
Isopropyl beta-D-glucoside
L-Malic acid
Ginkgoic acid
MG(0:0/22:6(4Z,7Z,10Z,13Z,16Z,19Z)/0:0)
Acetylcarnitine
Glutamylproline
Febuxostat
P-Tolyl Sulfate
O-Cresol
Indoxylsulfuric acid
Trigonellinamide
2-Oxo-4-methylthiobutanoic acid
Homolanthionine
(R)-3-hydroxybutyrylcarnitine
Corticosterone
LysoPC(20:4(5Z,8Z,11Z,14Z))
N-Acetylglutamine
C-2 Ceramide
15-cyclohexyl pentanor PGF2alpha
21-Deoxycortisol
DG(8:0/0:0/10:0)
Daphniphylline
N-Acetyl-D-phenylalanine
L-Histidinol
LysoPC(P-16:0)
CAY10444
Succinic acid
LysoPC(22:4(7Z,10Z,13Z,16Z))
Trihydroxycoprostanoic acid
8,11-eicosadiynoic acid
N-Acetyl-L-glutamic acid
PC(P-18:1(11Z)/18:2(9Z,12Z))
Furanodiene
Methylpyrazine
3b-Allotetrahydrocortisol
1-(11Z,14Z-eicosadienoyl)-glycero-3-phosphate
c
Vehicle
Mirabegron
Lipid metabolism
LysoPC(22:1(13Z))
Glycerylphosphorylethanolamine
11,14,17-Eicosatrienoic acid
Glycerophosphocholine
LysoPC(20:0/0:0)
LysoPC(20:1(11Z))
PE(14:0/22:2(13Z,16Z))
Sphinganine
Dodecanoic acid
LysoPC(22:4(7Z,10Z,13Z,16Z))
LysoPC(20:4(5Z,8Z,11Z,14Z))
Corticosterone
21-Deoxycortisol
d
FAO-related genes
Vehicle
Mirabegron
Ehhadh
Acox2
Acot12
Acad11
Acsm3
Acsl1
Acox1
Acsm5
Crot
max
min
e
FFA transporter-related genes
Vehicle
Mirabegron
Fabp1
Slc27a2
Slc27a5
Cd36
Fabp4
Fabp5
Cpt1a
Cpt1c
Cpt2
Slc27a4
max
min

Fig S5

Fig. S5 Unbiased metabolomic analysis in mirabegron-treated tumors

a. Principal component analysis of untargeted metabolomics data from vehicle- and mirabegron-treated PDAC tumors (n = 6 samples per group). Green dots, vehicle; Blue dots, mirabegron. **b.** Hierarchical clustering analysis and VIP scores of total metabolites in vehicle- and mirabegron-treated PDAC tumors (n = 6 samples per group). Red-blue color scale indicates the relative amount of metabolites expressed in each sample. VIP score was calculated based on the PLS-DA model. **c.** Heatmaps of the differential lipid metabolism-related metabolites (n = 6 samples per group). **d.** Heatmaps of fatty acid oxidation-related genes in vehicle- and mirabegron-treated tumors (n = 6 samples per group). **e.** Heatmaps of free fatty acid transporter-related genes in vehicle- and mirabegron-treated tumors (n = 6 samples per group). Metabolomic-related analysis (a-e) were performed once. Source data are provided as a Source Data file.

a

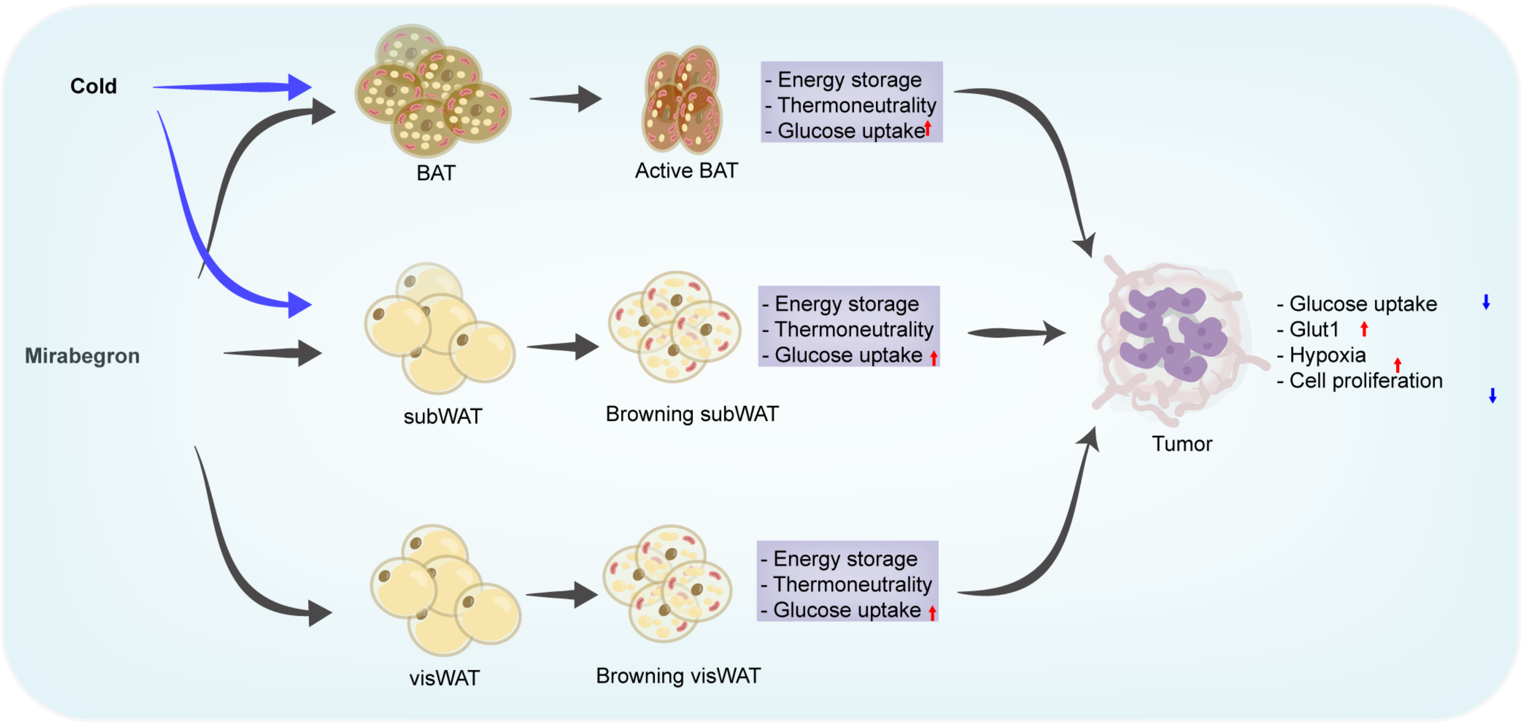

Fig S6

Fig. S6 Schematic diagram of mechanisms underlying mirabegron-suppressed tumor.

a. Cancer cells recklessly acquire their energy supply mainly through glycolysis i.e., the Warburg effect. During tumor development, adipose depots including BAT and WAT remain metabolically inert without significant glucose uptake and thermogenic activity. Under mirabegron treatment, BAT, subWAT, and visWAT undergo a robust browning process that markedly increases glucose uptake and thermogenesis. Of note, compared to cold-stimulation, mirabegron additionally activates visWAT which represents a significant amount of WAT. Thus, under the intervention of mirabegron, both WATs and BAT participate in glucose competition. Together, the glucose uptake pattern shifts toward adipose depots but not tumor, hence tumor growth is inhibited. Several possible mechanisms may be involved in mirabegron's anticancer effect: 1) reduced glucose supply in tumors; 2) trimmed glucose transportation in tumor cells; 3) reduced glucose metabolism in tumor cells; 4) alleviation of tumor hypoxia; 5) downregulated lipid metabolism in tumor cells. Source data are provided as a Source Data file.
