# Supplementary material for: Mirabegron displays anticancer effects by globally browning adipose tissues
Source: Nat Commun. 2023 Nov 22;14:7610. doi: 10.1038/s41467-023-43350-8 (PMC10665320; doi:10.1038/s41467-023-43350-8)
Supplement: Supplementary file 3 — Reporting Summary [file 41467_2023_43350_MOESM3_ESM.pdf]

nature portfolio

Corresponding author(s): Yihai cao

Last updated by author(s): Sep 27, 2023

Reporting Summary

Nature Portfolio wishes to improve the reproducibility of the work that we publish. This form provides structure for consistency and transparency in reporting. For further information on Nature Portfolio policies, see our Editorial Policies and the Editorial Policy Checklist.

Statistics

For all statistical analyses, confirm that the following items are present in the figure legend, table legend, main text, or Methods section.

n/a	Confirmed	
☐	☒	The exact sample size (*n*) for each experimental group/condition, given as a discrete number and unit of measurement
☐	☒	A statement on whether measurements were taken from distinct samples or whether the same sample was measured repeatedly
☐	☒	The statistical test(s) used AND whether they are one- or two-sided<br>*Only common tests should be described solely by name; describe more complex techniques in the Methods section.*
☐	☒	A description of all covariates tested
☐	☒	A description of any assumptions or corrections, such as tests of normality and adjustment for multiple comparisons
☐	☒	A full description of the statistical parameters including central tendency (e.g. means) or other basic estimates (e.g. regression coefficient) AND variation (e.g. standard deviation) or associated estimates of uncertainty (e.g. confidence intervals)
☐	☒	For null hypothesis testing, the test statistic (e.g. *F*, *t*, *r*) with confidence intervals, effect sizes, degrees of freedom and *P* value noted<br>*Give P values as exact values whenever suitable.*
☒	☐	For Bayesian analysis, information on the choice of priors and Markov chain Monte Carlo settings
☒	☐	For hierarchical and complex designs, identification of the appropriate level for tests and full reporting of outcomes
☒	☐	Estimates of effect sizes (e.g. Cohen's *d*, Pearson's *r*), indicating how they were calculated

Our web collection on statistics for biologists contains articles on many of the points above.

Software and code

Policy information about availability of computer code

Data collection	-Immunofluorescence images were captured using a fluorescence microscope (Olympus BX53, Japan) and quantified using Adobe Photoshop software CS5 extended (Adobe) and ImageJ Fiji.<br>- For H&E staining, the image was captured by a light microscope (Leica DM IL LED) and quantified using Adobe Photoshop software CS5 extended (Adobe) and ImageJ Fiji.<br>-The mass spectrometry metabolomics data were collected using Thermo UHPLC-Q Exactive Mass Spectrometer equipped with an electrospray ionization (ESI) source.<br>-PET-CT images were quantified using Inveon MicroPET/CT system includes Inveon Acquisition workplace and Inveon Research workplace software Version 4.2 (Siemens Medical Solution, California, USA).
Data analysis	Microsoft 365 Excel; GraphPad Prism 9.2.0;

For manuscripts utilizing custom algorithms or software that are central to the research but not yet described in published literature, software must be made available to editors and reviewers. We strongly encourage code deposition in a community repository (e.g. GitHub). See the Nature Portfolio guidelines for submitting code & software for further information.

Data

Policy information about availability of data

All manuscripts must include a data availability statement. This statement should provide the following information, where applicable:

- Accession codes, unique identifiers, or web links for publicly available datasets
- A description of any restrictions on data availability
- For clinical datasets or third party data, please ensure that the statement adheres to our policy

The metabolomics raw data generated in this study have been deposited in the MetaboLights database under accession code MTBLS868 (www.ebi.ac.uk/metabolights/MTBLS8682). Source data are provided with this paper.

Research involving human participants, their data, or biological material

Policy information about studies with human participants or human data. See also policy information about sex, gender (identity/presentation), and sexual orientation and race, ethnicity and racism.

Reporting on sex and gender	*Use the terms sex (biological attribute) and gender (shaped by social and cultural circumstances) carefully in order to avoid confusing both terms. Indicate if findings apply to only one sex or gender; describe whether sex and gender were considered in study design; whether sex and/or gender was determined based on self-reporting or assigned and methods used.*<br>*Provide in the source data disaggregated sex and gender data, where this information has been collected, and if consent has been obtained for sharing of individual-level data; provide overall numbers in this Reporting Summary. Please state if this information has not been collected.*<br>*Report sex- and gender-based analyses where performed, justify reasons for lack of sex- and gender-based analysis.*
Reporting on race, ethnicity, or other socially relevant groupings	*Please specify the socially constructed or socially relevant categorization variable(s) used in your manuscript and explain why they were used. Please note that such variables should not be used as proxies for other socially constructed/relevant variables (for example, race or ethnicity should not be used as a proxy for socioeconomic status).*<br>*Provide clear definitions of the relevant terms used, how they were provided (by the participants/respondents, the researchers, or third parties), and the method(s) used to classify people into the different categories (e.g. self-report, census or administrative data, social media data, etc.)*<br>*Please provide details about how you controlled for confounding variables in your analyses.*
Population characteristics	*Describe the covariate-relevant population characteristics of the human research participants (e.g. age, genotypic information, past and current diagnosis and treatment categories). If you filled out the behavioural & social sciences study design questions and have nothing to add here, write "See above."*
Recruitment	*Describe how participants were recruited. Outline any potential self-selection bias or other biases that may be present and how these are likely to impact results.*
Ethics oversight	*Identify the organization(s) that approved the study protocol.*

Note that full information on the approval of the study protocol must also be provided in the manuscript.

Field-specific reporting

Please select the one below that is the best fit for your research. If you are not sure, read the appropriate sections before making your selection.

☒ Life sciences ☐ Behavioural & social sciences ☐ Ecological, evolutionary & environmental sciences

For a reference copy of the document with all sections, see nature.com/documents/nr-reporting-summary-flat.pdf

Life sciences study design

All studies must disclose on these points even when the disclosure is negative.

Sample size	No statistical calculations were used to predetermine sample size for in vivo experiments. Sample sizes are indicated in Figure legends. Our previous tumor studies [1-3] determined the sample sizes by the extent and consistency of measurable differences to ensure statistical and biological significance. Besides, an optimal number of animals were employed for the aspect of the 3R principle for animal ethical permission [4].<br><br>[1] Xiaoting Sun et al. Inflammatory cell-derived CXCL3 promotes pancreatic cancer metastasis through a novel myofibroblast-hijacked cancer escape mechanism. Gut. 2022 Jan;71(1):129-147.<br>[2] Kayoko Hosaka et al, Therapeutic paradigm of dual targeting VEGF and PDGF for effectively treating FGF-2 off-target tumors. Nat Commun. 2020 Jul 24;11(1):3704.<br>[3] Hideki Iwamoto et al. Cancer lipid metabolism confers antiangiogenic drug resistance. Cell Metab. 2018 Jul 3;28(1):104-117.e5.<br>[4] Swedish 3R-center, https://jordbruksverket.se/languages/english/the-swedish-3rs-center
Data exclusions	No data were excluded.

Replication	Murine studies: Experiments were performed at least 3 times.All attempts at replication were successful. The fundamental tumor experiments for different drug treatment were performed at least 3 times and all attempts at replication were successful. Omics studies: Quality samples control was performed before subjecting those analyses. Analysis for metabolites for Fig. 4 was performed once using six independent biological replicants. For qPCR test:Experiments were performed at least 3 times independently and all attempts at replication were successful. Cell studies: Experiments were performed at least 3 times independently and all attempts at replication were successful.
Randomization	Murine studies: Age, gender, and background-matched mice were randomly allocated into the groups for all animal experiments.Omics studies: samples were randomly allocated into the groups. Cell studies: Cells were randomly allocated into the groups.
Blinding	Murine studies: Blinding was not performed for experiments involving different drug treatment because the color of drug was different. PET-CT technologist at Fudan University in China was blinded to the group allocation.<br>Omics studies: The authors who were blinded to experimental groups performed the metabolite analyses in mouse tissues.

Reporting for specific materials, systems and methods

We require information from authors about some types of materials, experimental systems and methods used in many studies. Here, indicate whether each material, system or method listed is relevant to your study. If you are not sure if a list item applies to your research, read the appropriate section before selecting a response.

Materials & experimental systems

n/a	Involved in the study
☐	☒ Antibodies
☐	☒ Eukaryotic cell lines
☒	☐ Palaeontology and archaeology
☐	☒ Animals and other organisms
☒	☐ Clinical data
☒	☐ Dual use research of concern
☒	☐ Plants

Methods

n/a	Involved in the study
☒	☐ ChIP-seq
☒	☐ Flow cytometry
☒	☐ MRI-based neuroimaging

Antibodies

Antibodies used

Information for antibodies for this study are,
- Rabbit anti-mouse Cleaved Caspase 3 polyclonal antibody (1:200; 9661; Cell Signaling)
- Rabbit anti-mouse Ki67 antibody (1:100; PA5-19462; Thermo Fisher Scientific)
- Rabbit anti-mouse polyclonal CA9 (1:100; NB100-417; Novus biologicals)
- Rabbit anti-mouse UCP1 polyclonal antibody (1:200; ab 10983; abcam)
- Rabbit anti-mouse COX4 polyclonal antibody (1:300; GTX114330, GeneTex)
- Guinea pig anti-mouse Perilipin antibody (1:300, 20R-PP004, Fitzgerald Industries)
- Alexa Fluor 555 goat anti-rabbit antibody (1:300; A21428, Thermo Fisher Scientific)
- Alexa Fluor 488-labeled goat anti-rabbit antibody (1:300, A11008, Thermo Fisher Scientific)
- Alexa Fluor 647 goat anti-guinea pig antibody (1:200; A-21450, Thermo Fisher Scientific)

Validation

All antibodies used in this study were validated for the application and species by their manufacturers. The link is listed below.
- Rabbit anti-mouse Cleaved Caspase 3 polyclonal antibody (1:200; 9661; Cell Signaling)
https://www.cellsignal.com/products/primary-antibodies/cleaved-caspase-3-asp175-antibody/9661
- Rabbit anti-mouse Ki67 antibody (1:100; PA5-19462; Thermo Fisher Scientific)
https://www.thermofisher.cn/cn/zh/antibody/product/Ki-67-Antibody-Polyclonal/PA5-19462
- Rabbit anti-mouse polyclonal CA9 (1:100; NB100-417; Novus biologicals)
https://www.novusbio.com/products/carbonic-anhydrase-ix-ca9-antibody_nb100-417
- Rabbit anti-mouse UCP1 polyclonal antibody (1:200; ab 10983; abcam)
https://www.abcam.com/ucp1-antibody-ab10983.html
- Rabbit anti-mouse COX4 polyclonal antibody (1:300; GTX114330, GeneTex)
https://www.genetex.com/Product/Detail/COX4-antibody/GTX114330
- Guinea pig anti-mouse Perilipin polyclonal antibody (1:300; 20R-PP004, Fitzgerald Industries)
https://www.fitzgerald-fii.com/perilipin-antibody-20r-pp004.html
-Alexa Fluor 555 goat anti-rabbit antibody (1:300; A21428, Thermo Fisher Scientific)
https://www.thermofisher.com/antibody/product/Goat-anti-Rabbit-IgG-H-L-Cross-Adsorbed-Secondary-Antibody-Polyclonal/A-21428
- Alexa Fluor 488-labeled goat anti-rabbit antibody (1:300, A11008, Thermo Fisher Scientific)
https://www.thermofisher.cn/cn/zh/antibody/product/Goat-anti-Rabbit-IgG-H-L-Cross-Adsorbed-Secondary-Antibody-Polyclonal/A-11008
- Alexa Fluor 647 goat anti-guinea pig antibody (1:200; A-21450, Thermo Fisher Scientific)
https://www.thermofisher.com/antibody/product/Goat-anti-Guinea-Pig-IgG-H-L-Highly-Cross-Adsorbed-Secondary-Antibody-

Polyclonal/A-21450

Eukaryotic cell lines

Policy information about cell lines and Sex and Gender in Research

Cell line source(s)	- Murine pancreatic cancer cell line Panc02 was kindly provided by Dr Maximilian Schnurr at the University of Munich, Germany<br>- Murine hepatocellular carcinoma cell line Hepa1-6 was purchased from ATCC (CRL-1830™).<br>- Murine MC-38 colon adenocarcinoma cell line was kindly provided by Dr. Rubén Hernández at the University of Navarra, Spain.
Authentication	All cell lines were not authenticated.
Mycoplasma contamination	All cell lines used in our study were negative for mycoplasma as detected by a mycroplasma kit (Cat. No. LT07-318, Lonza).
Commonly misidentified lines (See ICLAC register)	No commonly misidentified cell line was used.

Animals and other research organisms

Policy information about studies involving animals; ARRIVE guidelines recommended for reporting animal research, and Sex and Gender in Research

Laboratory animals	This study used laboratory mice for in vivo animal model.<br>Male 6-8-week-old wild type C57Bl/6 and C57Bl/6J background-UCP1-/-mice were obtained from GemPharmatech, China and maintained under a 12-hour dark/12-hour light cycle with food (SCXK2020-0004, Beijing Keao Xieli Feed) and water provided ad libitum. ; Male 9-10-week-old C57Bl/6J background-ApcMin/+ mice were obtained from the Jackson Laboratory. and maintained under a 12-hour dark/12-hour light cycle with food (SCXK2020-0004, Beijing Keao Xieli Feed) and water provided ad libitum
Wild animals	This project did not use wild animals.
Reporting on sex	The mice used in this study were all male. The mechanism of this study is independent of the sex of the mice, and males are used to make the results more comparable.
Field-collected samples	This study did not collect samples from the field.
Ethics oversight	All mouse studies were approved by the Animal Experimental Ethical Committee of Fudan University, Shanghai, China.

Note that full information on the approval of the study protocol must also be provided in the manuscript.
